# Label-free imaging to study phenotypic behavioural traits of cells in complex co-cultures

**DOI:** 10.1038/srep22032

**Published:** 2016-02-26

**Authors:** Rakesh Suman, Gabrielle Smith, Kathryn E. A. Hazel, Richard Kasprowicz, Mark Coles, Peter O’Toole, Sangeeta Chawla

**Affiliations:** 1 Phasefocus Ltd, Sheffield, UK; 2 Technology Facility, University of York, UK; 3 Centre for Immunology and Infection, York, UK; 4 Department of Biology, University of York, UK

## Abstract

Time-lapse imaging is a fundamental tool for studying cellular behaviours, however studies of primary cells in complex co-culture environments often requires fluorescent labelling and significant light exposure that can perturb their natural function over time. Here, we describe ptychographic phase imaging that permits prolonged label-free time-lapse imaging of microglia in the presence of neurons and astrocytes, which better resembles *in vivo* microenvironments. We demonstrate the use of ptychography as an assay to study the phenotypic behaviour of microglial cells in primary neuronal co-cultures through the addition of cyclosporine A, a potent immune-modulator.

Observing cells over time utilising either brightfield or fluorescence based techniques is a valuable tool to study cellular behaviour in co-culture models^1^. Whilst brightfield microscopy does not require cell labelling, the suitability of this approach in mixed cell co-cultures is limited by lack of contrast and presence of imaging artefacts making it difficult to easily distinguish different cell types. Fluorescence labelling of living cells is therefore frequently used to overcome these limitations, where cells are either labelled using organic dyes or as expression of fluorescently encoded reporter genes. Although fluorescence labelling provides the contrast needed to differentiate between cells it has the potential to alter normal cell function. Furthermore extended time-lapse imaging of fluorescently labelled cells can cause significant phototoxicity^2^, which limits the duration of continuous imaging. Focal drift is an additional problem associated with prolonged time-lapse imaging as specimen movement from the focal plane can result in experimental failure as images become uninterpretable. Although autofocusing hardware and algorithms are available, these require additional light exposure and are susceptible to culture artefacts. To overcome these limitations, there has been a steady rise in the development of label-free imaging platforms that exploit phase shifted information from specimens^3^,^4^,^5^,^6^,^7^,^8^. These label-free technologies are ideally suited to cell cultures and stem cells where labelling of cells may be undesirable or not possible (e.g. primary human cells). We demonstrate the use of ptychography as a novel label-free imaging assay that permits continuous and prolonged time-lapse imaging of complex primary co-cultures. Ptychography also allows virtual post-acquisition focusing, preventing loss of images due to focal drift during image collection.

Through the use of an iterative phase retrieval algorithm, ptychography generates label-free, high contrast quantitative phase images^3^. In this technique the specimen is moved through a coherent illumination probe yielding an array of overlapping diffraction patterns that are recorded onto a CCD camera. The collected diffraction patterns are then processed by the ptychographic algorithm to produce an image that has both amplitude and phase information that relate to the refractive index and the thickness of the specimen^3^. Recently, ptychography has been used to quantify key cellular and biological events such as mitosis, cell cycle^6^ and angiogenesis^9^. In these studies, ptychographic imaging only required very low illumination power densities in the region of pW/μm^2^ at 635 nm, which permits the near continuous imaging of cells over many days.

## Results and Discussion

Previously we demonstrated the advantages of ptychography to image commonly used immortalised cell lines^6^. Here we reveal a new application of ptychography for long-term imaging of complex primary neuronal cultures^10^, where phenotypic behaviour of microglia can be studied. Microglia are the resident macrophages of the mammalian brain where they exhibit either a surveillance role^11^,^12^ in the healthy brain or respond to neurological insults by adopting an activated state^13^. In many studies immortalised microglial cell lines are used to examine the function of activated microglia^14^. However, the use of such cell lines lacks the contextual environment within which therapeutics for treating neurological conditions would need to act to be effective *in vivo*. Microglia behaviour has been shown to be dynamically linked to interactions with both neurons^15^ and astrocytes^16^. Neurons in particular are able to provide on or off signals that can manipulate microglial activity^15^ and conversely microglia have been shown to modulate neuronal plasticity^17^. Here we have developed and demonstrated the potential of a novel phenotypic assay using ptychography for label-free imaging of primary microglia activity/behaviour over prolonged time periods in the presence of both astrocytes and neurons.

**Figure 1:**
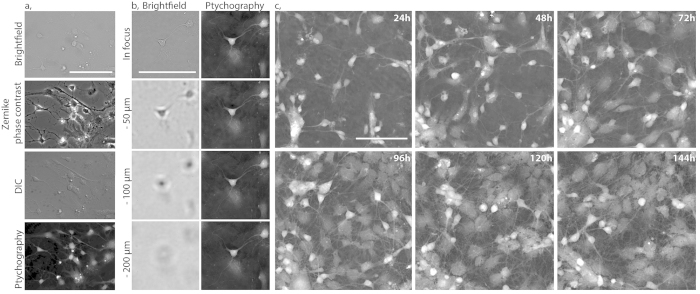
Time-lapse ptychographic phase imaging of primary rat hippocampal neuronal cultures. **(a**) Brightfield, Zernike phase contrast, DIC and ptychographic phase image showing the same field of view of primary neuronal cultures. (**b**) Brightfield images captured on the VL21 microscope acquired at various focal positions by moving objective in the z axis. At each focal position a ptychographic phase image was obtained and successfully refocused post-acquisition. (**c**) Maturation of primary hippocampal neuron cultures imaged in time-lapse starting at day 1 *in-vitro*. A 550 × 550 μm field of view was acquired every 6 minutes for a total period of 6 days (144 hours). Representative images show 24 hour cropped snapshots from a continuous time lapse sequence (scale bar = 100 μm, full time lapse sequence available as supplementary video S1).

We began by comparing the capability of ptychography and established label-free imaging techniques to image cells in a co-culture environment. Differences between ptychographic phase and classic bright field imaging, are highlighted in Fig. 1a; although cells could be identified in all cases, brightfield (BF)/differential interference contrast (DIC) and phase contrast microscopy possess inherent problems and artefacts that undermine their suitability to image and track cells over time. Brightfield microscopy, as expected, lacked sufficient contrast to discern cellular details and cellular boundaries were not readily identified. DIC similarly struggled with the grey levels varying relatively little between different cell types. Imaging using Zernike phase contrast effectively displayed the neurites, however it suffered from the classic halo artefacts around neuronal cell soma, which can become detrimental at higher cell densities or where cells are in close proximity. Imaging of primary neuronal cultures using ptychography offered superior contrast without generating imaging and post-processing artefacts, enabling cellular identification even when cells were in direct contact in the co-cultures.

Maintaining the specimen in the focal plane is a crucial requirement for successful time-lapse imaging. We tested the post-acquisition refocusing capability of ptychography by defocusing the specimen by 50, 100 and 200 μm (Fig. 1b). Brightfield images of the sample when moved 50 μm from the imaging plane are incomprehensible. Strikingly, due to its inherent post-acquisition refocusing capability, ptychographic phase images taken at the same z positions as the brightfield images maintain contrast and quality.

We next sought to test the non-invasive properties of ptychography by imaging dissociated primary hippocampal cultures in prolonged time-lapse experiments. A large (550 × 550 μm) field of view of the primary cultures starting at day one *in vitro*, was imaged every six minutes for six days (Fig. 1c). Over the duration of the time-lapse, maturation of these cultures can be seen clearly as the hippocampal neurons develop extensive neurite connectivity together with the proliferation of both microglia and astrocytes (supplementary video S1).

**Figure 2:**
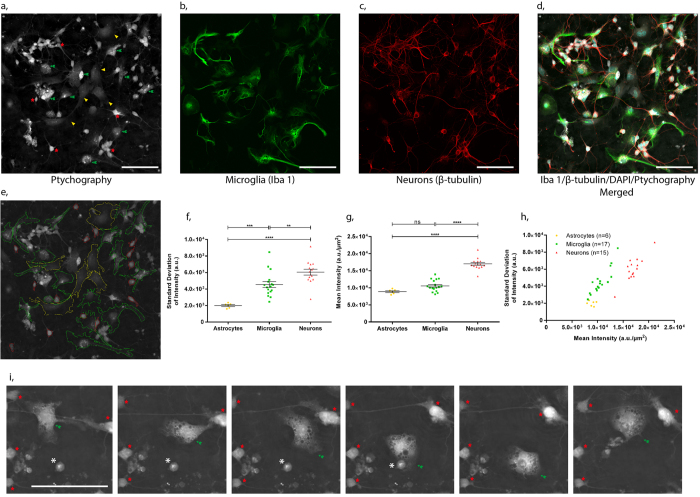
Correlative microscopy and quantitative analysis of ptychographic phase images. **(a**) Ptychographic phase image of the live primary hippocampal culture was acquired prior to fixation, where microglia (green arrows), neurons (red stars) and astrocytes (yellow triangles) are easily distinguished. Following fixation correlative confocal images from the same field of view show (**b**) microglia labelled with Iba1, (**c**) hippocampal neurons labelled with β-tubulin and (**d**) a merged image of Iba1, β-tubulin, DAPI labelled cells overlaid with the ptychographic phase image. The remaining unlabelled population of cells represent astrocytes in the culture. (**e**) Segmentation of the individual cell types (microglia-green, neurons-red and astrocytes-yellow). (**f**) Standard deviation of pixel intensity, (**g**) mean intensity and (**h**) and the relationship between the two parameters for each segmented cell (one way ANOVA with Tukey’s post-hoc test, ****p < 0.0001, ***p < 0.001, **p < 0.01). (**i**) Ptychographic phase images from a time-lapse sequence clearly showing the phagocytic activity of microglia (indicated by a green arrow) towards an apoptotic neuron (indicated by an asterisk). Healthy neurons are indicated by a red star. Further examples of phagocytic activity in supplementary video S2.

Having demonstrated the long-term label-free imaging capability of ptychography, we next investigated the extent to which individual cell types within the culture could be distinguished. To achieve this a hippocampal culture specimen was imaged using ptychography (Fig. 2a) followed by confocal microscopy of the identical field of view, where cells were fluorescently labelled with antibodies raised against microglia (Fig. 2b) or neurons (Fig. 2c). Hippocampal neurons were easily identified due to their distinct morphology in the ptychographic phase images. The cell soma were small, yet their phase shift was relatively large due to their thickness giving the cells a unique bright characteristic in the ptychographic phase images. Neurites were also easily distinguishable against the background. Conversely astrocytes were relatively thin and therefore did not appear as bright in terms of phase intensity. The morphology of the astrocytes and microglia in the culture was also very different from the hippocampal neurons; whilst the shape of the microglia was similar to that of astrocytes, they differed greatly with regards to intracellular morphology. Microglial cells have a granular cytoplasmic structure and often appear vacuolated.

Quantitative analysis was then undertaken on the segmented cells of the ptychographic phase image shown in Fig. 2e. Here we measured the standard deviation of pixel intensity and the mean intensity within each cell. Microglia, astrocytes and neurons were individually distinguished based on these two parameters (Fig. 2e–h) where the individual cell types had a clear subset of characteristics. Microglia displayed a significantly higher standard deviation of pixel intensity (4.55 × 10^3^ ± 361) than astrocytes (1.20 × 10^3^ ± 117), although their mean pixel intensity was similar (10.6 × 10^3^ ± 407, 8.89 × 10^3^ ± 274, respectively). This likely reflects the differences in intracellular granularity between the cell types. Neurons on the other hand displayed a significantly higher mean intensity (17.0 × 10^3^ ± 426) than either microglia (10.6 × 10^3^ ± 407) or astrocytes (8.89 × 10^3^ ± 274).

Furthermore by viewing the culture in time-lapse the individual cells types could be identified due to their motion and behaviour. Microglia cells are very motile in comparison to astrocytes and also exhibited characteristic phagocytic activity (Fig. 2i and supplementary video S2). This ability to distinguish the individual cells without labels provides a platform to assess microglial cell behaviour and activity. The dynamic interplay that exists between the three cell types^15^,^16^,^17^ clearly identifies a requirement to study them in co-cultures without the introduction of labels that have the potential of perturbing the underlying cell behaviours.

To exploit the ability of long-term imaging together with the capability to identify individual cell types within neuronal cultures, we assessed biologically relevant aspects of microglial behaviour and its pharmacological modulation. Cyclosporine A (CsA) has previously been reported to reduce microglia activation and activity using biochemical and immuno-fluorescent assays^18^,^19^. Here we investigated the effects of CsA on phenotypic traits of microglia *in vitro*, without the addition of any fluorescent labels and therefore monitoring their activity in an un-perturbed system.

**Figure 3:**
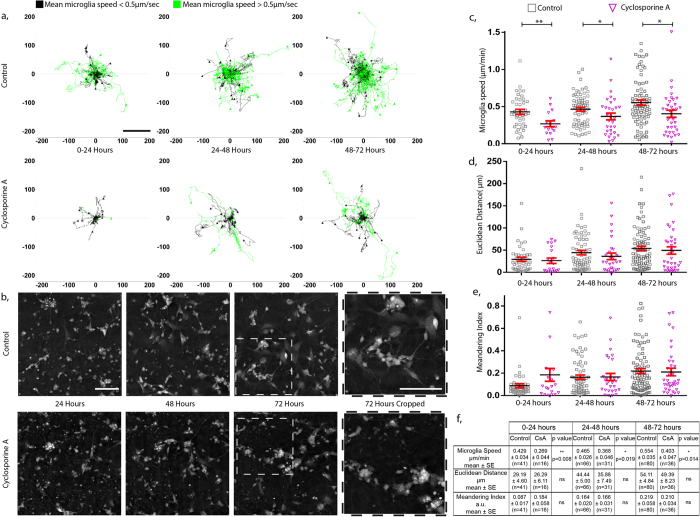
Quantifying and measuring microglia activity. **(a**) Activity of microglia from 3 independent rodent neuronal cultures was measured over a period of 72 hours following treatment with 1 μM cyclosporine A and compared to control conditions. Tracks of individual microglia are plotted from their point of origin (μm) in each 24 hour window over the 72 hour period. Tracks in black represent microglia that possess a mean speed that is less than 0.5 μm/min whilst those in green have a mean speed greater than 0.5 μm/min. (**b**) Representative images at each 24 hour time point show the development and the maturation of both control and cyclosporine A treated neuronal cultures (full time-lapse videos available as supplementary videos S3 and S4). Quantitative parameters relating to the microglia activity such as; (**c**) speed; (**d**) Euclidean distance and (**e**) meandering index were calculated for multiple microglia from both the control and cyclosporine A treated neuronal cultures during each time period. (**f**) These values are summarised in a table form (Mann-Whitney non-parametric test of significance, **p < 0.01, *p < 0.05).

Following application of 1 μM CsA, microglia motility was tracked in the time-lapse videos over a 72 hour period. The effects of CsA on microglial speed, Euclidean distance and meandering index was calculated for each 24-hour time-window over the 72 hours of continuous imaging. Microglia tracks are presented as migration plots from their point of origin at each time window (Fig. 3a). The application of 1 μM CsA significantly reduced microglia speed by 32.3%, 20.7% and 27.2% at the time points of 0–24, 24–48 and 48–72 hours, respectively when compared to controls (Fig. 3c,f). However, CsA had no effect on the Euclidean distance (Fig. 3d,f) or meandering index (Fig. 3e,f). Moreover, cultures treated with CsA for 72 hours (Fig. 3b) displayed evidence of more cellular debris and the neuronal processes were less pronounced. Taken together, our data indicate that CsA reduces microglia speed without affecting the size of the domain within which they migrate. This reduction in speed defines a novel phenotypic trait of microglial behaviour that is influenced by CsA; this is in addition to the already established effects of CsA on cytokine release^19^. Such behavioural traits could be used for low or medium throughput screens for candidate compounds that could be used to treat neurological conditions^14^.

Cells in culture and in particular co-culture are heterogeneous in nature, and averaging these populations in assays undermines the properties of subpopulations^20^,^21^. Imaging studies are often limited to end-point assays that remove critical behavioural information. Here we have demonstrated the use of ptychography for prolonged time-lapse imaging of multiple cell types in co-cultured environments, revealing behavioural and phenotypic information of microglia.

The use of ptychography in the present study offers significant advantages over conventional label-free imaging techniques such as Zernike phase contrast microscopy. The superior contrast alone enables greater confidence and robustness in tracking the microglia, especially in confluent complex cell cultures (Fig. 1a). Ptychography also has the inherent ability to re-focus images post acquisition, without the need for additional autofocusing hardware (Fig. 1b). This capability is crucial to overcome focal drift when performing time-lapse imaging studies. Furthermore, ptychography offers quantitative outputs that relate to physical properties of the cell itself (Fig. 2e–h), thus demonstrating the future applicability of this type of imaging to quantitatively differentiate between multiple cell types. Taken together, these features are a notable advantage over conventional label-free imaging.

We anticipate that the label-free, non-invasive and quantitative features of ptychography will be widely applicable across many live-cell imaging studies providing subtle behavioural and phenotypic information that has not before been studied.

## Methods

### Primary hippocampal cell culture and preparation

Mixed cultures of hippocampal neurons, astrocytes and microglia were prepared from new born Wistar rats as described in Belfield *et al.* (2006)^10^ except that the growth medium was Neurobasal medium (Invitrogen, Carlsbad, CA) containing 2% B27 (Invitrogen), 5% foetal calf serum (PAA Lab, Pasching, Austria), 1 mM L-glutamine, 35 mM glucose (Sigma, St. Louis, MO), 100 U/ml of penicillin, and 0.1 mg/ml streptomycin (Invitrogen).

Cells were plated in poly-l-lysine treated 8 well Lab-Tek II chambered coverslips (Nalge Nunc Int, Rochester, NY). Immediately prior to imaging the wells were filled with conditioned media and sealed with a coverslip (22 × 50 mm, no. 1.5, SLS, Hessle, UK) and high vacuum grease (Dow Corning Corp, Midland, MI). This eliminated the meniscus and provided an airtight seal so that only the temperature was to be maintained.

### Ptychographic phase imaging

Ptychographic phase images were acquired as described previously^6^, however in the current study we used the inverted VL21 microscope (Phasefocus, Sheffield, UK). Briefly the specimen was moved in the x-y plane through the 635 nm diode laser illumination. The resulting diffraction pattern was collected using a 16-bit, 1megapixel Pike AVT (F-100B) CCD camera set in the detector plane. An Olympus LMPlanFLN 20x/0.40 objective was used to collect the exit wave leaving the specimen.

Time-lapse imaging was performed by placing the chamber slide on the VL21 microscope surrounded by a Solent Scientific environmental chamber (Solent Scientific Limited, UK) maintaining the cells and the microscope at 37 °C. A 22 × 22 tile scan was selected that generated 484 overlapping diffraction patterns through movement of the x-y stage, with an acquisition time of approximately 90 s. Images were acquired in this manner by sequentially visiting 4 wells of the chamber slide with a time of 6 minutes between imaging the same well. Image reconstruction was performed post acquisition using the extended Ptychographic Iterative Engine (ePIE) algorithm (The Phase Focus Virtual Lens®, Phasefocus, UK, www.phasefocus.com). Time-lapse video sequences and 16-bit Tiff images were created using the Cell Analysis Toolbox (CAT) software package (Phasefocus, UK, www.phasefocus.com).

Correlative DIC and Zernike phase contrast images were acquired using Zeiss LSM 510 confocal microscope equipped with a Zeiss AxioCam HRm CCD camera and Axiovision software.

### Correlative confocal microscopy

Neurons were fixed in 3% paraformaldehyde in PBS supplemented with 4% sucrose and processed for immunocytochemistry as described in Belfield *et al.* (2006). The following primary antibodies were used at the indicated dilutions. Anti-Iba1 rabbit polyclonal antibody (Wako Chemicals, USA) was used at 1:200 to detect microglial cells. Anti-β-tubulin III (TuJ1) mouse monoclonal antibody (Sigma, St Louis, MO) was used at 1:500 to detect neurons. Anti-GFAP raised in guinea pig (Advanced Immunochemical, Long Beach, CA) was used at 1:200 to detect astrocytes. Images were acquired using a Zeiss LSM 780 confocal microscope.

### Quantitative analysis of ptychographic phase images

Ptychographic phase images were imported into ImageJ(Fiji), where manual segmentation was performed. The standard deviation of pixel intensity and mean intensity were measured for each segmented cell.

### Cell tracking and analysis

Time-lapse videos were generated using the CAT software (Phasefocus, UK) and loaded in to ImageJ(Fiji). The MtrackJ plugin was then used to track individual microglia over the 72 hour time-lapse. Quantitative cell motility parameters such as speed, meandering index and Euclidean distance were measured. Briefly the mean speed was calculated by dividing the total distance travelled by the microglia by the total time of the track. The meandering index was calculated as the final displacement of the microglia from the point of origin divided by the total distance travelled by the microglia. The Euclidean distance was calculated as the final displacement in a straight line from the point of origin of the microglia. The data was presented as rose-plots by importing the tracking co-ordinates into the Chemotaxis tool software (Ibidi GmbH, Germany).

### Statistical analysis

Data are represented as mean values ± SEM. Statistical significance was determined using one way ANOVA with Tukey’s post-hoc test or Mann-Whitney non-parametric test of significance, where appropriate. All statistical analysis was performed using Graphpad Prism Version 6.0.

## Additional Information

**How to cite this article**: Suman, R. *et al.* Label-free imaging to study phenotypic behavioural traits of cells in complex co-cultures. *Sci. Rep.* **6**, 22032; doi: 10.1038/srep22032 (2016).

## Supplementary Material

Supplementary Information
