# Supplementary material for: Label-free imaging to study phenotypic behavioural traits of cells in complex co-cultures
Source: Sci Rep. 2016 Feb 26;6:22032. doi: 10.1038/srep22032 (PMC4768090; doi:10.1038/srep22032)
Supplement: Supplementary Information [file srep22032-s1.doc]

**Supplementary Information for Suman et al., “Label-free imaging to study phenotypic behavioural traits of cells in complex co-cultures.”**

Rakesh Suman^1,2*^, Gabrielle Smith^3^, Kathryn E. A. Hazel^3^, Richard Kasprowicz^1^ Mark Coles^3,4^, Peter O’Toole^2,4^, Sangeeta Chawla^4*^

1 Phasefocus Ltd, Sheffield, UK

2 Technology Facility, University of York, UK

3 Centre for Immunology and Infection, York, UK

4 Department of Biology, University of York, UK

* Correspondence should be addressed to RS ([rakesh.suman@york.ac.uk](mailto:rakesh.suman@york.ac.uk)) or SC (sangeeta.chawla@york.ac.uk)

**Supplementary Video S1 - Long term (6-days) time-lapse imaging of primary hippocampal neuronal cultures.**

Maturation of primary hippocampal neuron cultures imaged in time-lapse starting at day 1 in-vitro. A 550 x 550 µm field of view was acquired every 6 minutes for a total period of 6 days (144 hours).

**Supplementary Video S2 - Phagocytic activity of microglia**

Time-lapse video demonstrates the phagocytic activity of microglia towards apoptotic bodies and cellular debris.

**Supplementary Video - 72 hour time-lapse (Control) of primary hippocampal neuronal cultures**

Time-lapse imaging of neuronal cultures under control show good neuronal development with microglia effectively clearing cellular debris and apoptotic material.

**Supplementary Video S4 - 72 hour time-lapse of cyclosporine A (1 μM) treated primary hippocampal neuronal cultures**

Time-lapse imaging of neuronal cultures treated with 1µM cyclosporine A, show restricted development of neuronal processes with and abundance of cellular debris.
